# Immunogenicity of HPV prophylactic vaccines: Serology assays and their use in HPV vaccine evaluation and development

**DOI:** 10.1016/j.vaccine.2017.11.089

**Published:** 2018-08-06

**Authors:** Ligia A. Pinto, Joakim Dillner, Simon Beddows, Elizabeth R. Unger

**Affiliations:** a Vaccine, Cancer and Immunity Program, Frederick National Laboratory for Cancer Research, Leidos Biomedical Research, Inc., Frederick, MD, USA; b Department of Laboratory Medicine, Karolinska Institutet, 141 86 Stockholm, Sweden; c Virus Reference Department, Public Health England, London, UK; d Chronic Viral Diseases Branch, Division of High-Consequence Pathogens and Pathology, National Center for Emerging and Zoonotic Infectious Diseases, Centers for Disease Control and Prevention, Atlanta, GA, USA

**Keywords:** Immunogenicity, Prophylactic HPV L1 VLP vaccines, Serology assays

## Abstract

When administered as standard three-dose schedules, the licensed HPV prophylactic vaccines have demonstrated extraordinary immunogenicity and efficacy. We summarize the immunogenicity of these licensed vaccines and the most commonly used serology assays, with a focus on key considerations for one-dose vaccine schedules.

Although immune correlates of protection against infection are not entirely clear, both preclinical and clinical evidence point to neutralizing antibodies as the principal mechanism of protection. Thus, immunogenicity assessments in vaccine trials have focused on measurements of antibody responses to the vaccine. Non-inferiority of antibody responses after two doses of HPV vaccines separated by 6 months has been demonstrated and this evidence supported the recent WHO recommendations for two-dose vaccination schedules in both boys and girls 9–14 years of age. There is also some evidence suggesting that one dose of HPV vaccines may provide protection similar to the currently recommended two-dose regimens but robust data on efficacy and immunogenicity of one-dose vaccine schedules are lacking. In addition, immunogenicity has been assessed and reported using different methods, precluding direct comparison of results between different studies and vaccines. New head-to-head vaccine trials evaluating one-dose immunogenicity and efficacy have been initiated and an increase in the number of trials relying on immunobridging is anticipated. Therefore, standardized measurement and reporting of immunogenicity for the up to nine HPV types targeted by the current vaccines is now critical. Building on previous HPV serology assay standardization and harmonization efforts initiated by the WHO HPV LabNet in 2006, new secondary standards, critical reference reagents and testing guidelines will be generated as part of a new partnership to facilitate harmonization of the immunogenicity testing in new HPV vaccine trials.

## 1 Current licensed HPV prophylactic vaccines

There are currently three licensed HPV prophylactic vaccines: Cervarix®, a bivalent HPV-16/18 product from GlaxoSmithKline; Gardasil®, a quadrivalent HPV-6/11/16/18 product and Gardasil®9, a nonavalent HPV-6/11/16/18/31/33/45/52/58 vaccine, both from Merck & Co., Inc. [1], [2], [3], [4]. These products were licensed following highly encouraging efficacy data from large phase III vaccine trials and have the potential (nonavalent vaccine) to prevent up to 90% of cervical cancer cases. The first two products to be licensed, Cervarix® and Gardasil®, used a placebo arm and relied on cervical disease as the primary endpoints [5], [6], [7]. The Gardasil®9 trial used the previously licensed Gardasil® vaccine as the control arm [8]. For the 4 HPV types targeted by both vaccines, the primary endpoint was non-inferiority in antibody response, while cervical disease endpoints were used for the 5 additional types in Gardasil®9. All the vaccine trials have demonstrated outstanding long-term efficacy. This remarkable vaccine efficacy is now starting to be seen as effectiveness at the population level following introduction of HPV vaccines into national immunization programs [9].

All three vaccines are based on non-infectious recombinant type-specific L1 capsid proteins assembled into viral-like particles (VLPs) as immunogens. The expressed recombinant L1 capsids self-assemble in arrays of 72 pentamers that present an exterior surface closely mimicking HPV virions and it is this multiplicity of L1 domains that bestows the VLP antigen with significant immunogenicity, even in the absence of concomitant immune modulators (adjuvants) [10], [11].

The vaccines differ in the antigen expression system used, antigen composition and adjuvants included (Table 1) [12], [13], [14]. Both Gardasil® and Gardasil®9 products are produced in yeast and formulated with amorphous aluminum hydroxyphosphate sulfate (AAHS) adjuvant, which has an increased capacity to bind to L1 VLPs compared with other aluminum salts [15]. In contrast, Cervarix® is produced in insect cells using a baculovirus expression vector system and adjuvanted with AS04 which contains aluminum hydroxide plus an additional immunostimulant, the toll-like receptor 4 agonist monophosphoryl lipid A [16]. AS04 has been shown to enhance innate as well as humoral and cell-mediated immune responses [17], and may be responsible for differences in the overall immunogenicity described in head-to-head studies of the two vaccines [18], [19], [20], [21]. Other differences between the vaccines include the concentration of each of the L1 VLPs, and the ratio of antigen to adjuvant. Gardasil® has two-fold higher concentrations of HPV-16 L1 VLP and an equal concentration of HPV-18 L1 VLP compared with Cervarix®. Gardasil®9 contains twice the amount of HPV-18 L1 VLP, 50% more HPV-16 antigen and more than twice the level of adjuvant contained in Gardasil®.

**Table 1:** Main characteristics of the licensed HPV prophylactic vaccines.

	Cervarix®	Gardasil®	Gardasil9®
Manufacturer	GlaxoSmithKline	Merck and Co, Inc.	Merck and Co, Inc.
VLP Types Included	HPV-16/18	HPV-6/11/16/18	HPV-6/11/16/18/31/33/45/52/58
Dose of L1 VLP (μg)	20/20	20/40/40/20	30/40/60/40/20/20/20/20/20
Expression system	*Trichoplusia ni* (Hi 5) insect cell line infected with L1 recombinant baculovirus	*Saccharomyces cerevisiae* expressing L1	*Saccharomyces cerevisiae* expressing L1
Adjuvant	500 μg aluminum hydroxide salt and 50 μg 3-O- Desacyl-4′-monophosphoryl lipid (MPL) A	225 μg amorphous aluminum hydroxyphosphate sulfate	500 μg amorphous aluminum hydroxyphosphate sulfate
Initially approved injection schedule	0, 1 and 6 months	0, 2, and 6 months	0, 2, and 6 months
Manufacturing components	4.4 mg NaCl, 0.624 mg sodium dihydrogen phosphate dihydrate	9.56 mg NaCl, 0.78 mg L-Histidine, 50 μg Polysorbate 80, 35 μg Sodium borate	9.56 mg NaCl, 0.78 mg L-Histidine, 50 μg Polysorbate 80, 35 μg Sodium borate
Route of administration	Intramuscular	Intramuscular	Intramuscular

The vaccines were originally licensed for use as a three-dose regimen, administered intramuscularly. The licensed HPV vaccines have demonstrated remarkable efficacy in phase III clinical trials in HPV-naïve young women, providing nearly complete protection against incident infection and cervical disease caused by the HPV types that they specifically target [5], [6], [7], [8], [22]. This is coincident with the induction of a high level, high affinity polyclonal anti-L1 IgG antibody response to the HPV types included in the vaccine, and essentially 100% seroconversion to all targeted HPV types [23], [24], [25], [26], [27], [28]. The robust immunogenicity of the HPV vaccines contrasts with the immune responses observed after natural infection, in which seroconversion is found in only a proportion of individuals following incident infection [29]. Vaccine antibodies persist for several years after vaccination at levels that are orders of magnitude higher than those observed in natural infection [24], [30], [31], [32]. Antibodies generated during natural infection do not appear to consistently protect against subsequent infection. In addition, some studies suggest that protection may be limited to individuals with relatively high levels of naturally-acquired antibody [33], [34]. These findings are consistent with a recent study indicating that antibodies cloned from naturally elicited memory B cells were generally non-neutralizing while those isolated after vaccination had strong neutralization activity [35].

A degree of efficacy against some non-vaccine types (i.e. cross-protection) has been demonstrated in vaccine trials, particularly for HPV-31 and HPV-45 [36], [37]. Cervarix® appears to confer greater cross-protection than Gardasil® and this difference is reflected in antibody levels against these non-vaccine types [38]. For the purposes of this review, however, only antibody responses against vaccine-targeted types are considered.

Correlates of protection against infection afforded by HPV vaccines have not been formally identified and are difficult to study because of the exceptional efficacy of the vaccines. There have been very few cases of breakthrough infection or disease with the current vaccines to identify the threshold of antibody level that confers immunity among vaccinated subjects. Most studies attempting to address this question have analyzed the protective levels of naturally acquired antibodies in the placebo arm of vaccine trials [33], [34] or in cohort studies of infection [29]. Evaluation of different immunogenicity measures in reduced dose schedule trials, in which virological failures may be more likely to occur, could make it possible to establish immune correlates of protection.

Immunogenicity testing of prophylactic HPV vaccines in clinical trials has focused primarily on antibody responses because neutralizing antibodies are thought to be the principal effectors of protection against HPV infection. This is primarily based on experimental evidence in animal models demonstrating that protection against papillomavirus infection can be passively transferred in serum or purified immunoglobulin (Ig) G from VLP-vaccinated animals [39]. It has also been shown that very low levels of HPV antibodies are able to neutralize HPV-16; the 50% inhibitory concentration being estimated to range from 1.9 picomolar to 5.4 nM for three monoclonal antibodies [40]. Vaccine-induced antibodies against vaccine types are detectable not only in serum but also at mucosal sites of infection, such as the cervix and oral cavity [41], [42], [43], [44]. These antibodies are believed to reach the site of infection through transudation from serum and by exudation at sites of potential trauma that expose the basement membrane to infection [43], [45].

## 2 Review of immunogenicity of licensed HPV vaccines and duration of responses

### 2.1 Immunogenicity of three-dose schedules

A number of recent publications have thoroughly reviewed the immunogenicity and efficacy of licensed HPV vaccines in the context of the standard three-dose and reduced dose regimens [46], [47]. While all three licensed vaccines have similar efficacy against HPV infection and precancer lesions in clinical trials, the products do differ in immunogenicity, as demonstrated in a variety of assays. Head-to-head trials of three doses of Cervarix® and Gardasil® in 18–45 year-old women and in 12–15 year-old girls have found that HPV-16 antibody levels were significantly lower for Gardasil® when compared with Cervarix® [19], [20], [21], [48], although they had similar patterns of peak and decay over time. HPV-18 antibody levels and seropositivity were significantly lower for Gardasil® than Cervarix®. Furthermore, Gardasil® induced lower HPV-16 and HPV-18 specific CD4^+^ T cell responses, as well as lower memory B cell responses, particularly for HPV-18 up to 24 months after vaccination [19], [20], [21].

High and durable HPV-16 and HPV-18 antibody levels, significantly above natural infection levels, have been reported after vaccination with Cervarix® for at least 9.4 years [30]. Stable HPV-16 antibody levels, above natural infection levels were described for Gardasil® for at least 9 years [24]. However, HPV-18 antibody levels appear to decline to levels closer to natural infection. The observed seropositivity for HPV-18 is assay dependent (See Section 3) and was found to be 91% with a total IgG binding Luminex immunoassay and 60% with competitive Luminex immunoassays (cLIA) in women 9 years after vaccination.

Gardasil®9 has been reported to elicit comparable HPV-16 and HPV-18 antibody responses as Gardasil®. HPV-18 antibody levels induced by three doses of Gardasil®9 measured with the cLIA also declined over time and 78% seropositivity was observed 5 years after vaccination [49]. A similar decline in HPV-45 seropositivity was described, while antibody seropositivity for other types was maintained.

Covariates of immunogenicity have been evaluated both for Gardasil® and Gardasil®9 in three dose recipients [50], [51]. Age at the time of immunization was inversely correlated with antibody levels at month 7 for all the 9 HPV types. For all 9 HPV types, vaccine-induced antibody levels were higher in girls and boys than in young women. Boys had slightly higher HPV antibody levels than girls of the same age. Overall, immunogenicity was generally comparable across different races and different geographical regions. Small differences in month 7 HPV antibody levels were observed in 16–26 year-old women of different races. Black women had slightly higher HPV antibody levels than Asian or white women or women of other races. Individuals in Africa, Latin America and North America tended to have higher HPV antibody levels than those in Asia, and Europe. In addition, pre-existing HPV antibodies at the time of vaccination resulted in higher HPV responses to that type.

Critical questions that will need to be addressed by ongoing and planned trials include whether higher vaccine-induced antibody levels would predict longer duration of protection and how to improve sensitivity and specificity of antibody assays so that reliable correlations can be made between protection against infection and detection of antibodies.

### 2.2 Immunogenicity of reduced dose schedules

Clinical studies evaluating reduced dose schedules and the interval between doses for both vaccines have demonstrated non-inferior antibody responses in girls younger than 15 years of age that received two doses, given six months apart, when compared with women who received the standard three doses of vaccine and had evidence of efficacy in clinical trials [52], [53], [54]. These findings have led to the recommendations and approval of two-dose schedules in 9–14 year-old girls [55]. Both Cervarix® and Gardasil® induced similar antibody levels following two and three vaccine doses, for their corresponding vaccine, when the doses were administered six months apart, in 18–25 year-old women as well as in 10–18 year-old girls, over 4 years of follow-up [56], [57]. Administration of two doses of Cervarix® 1 month apart resulted in lower antibody levels than two doses (given 6 months apart) or three doses, but they were stable over a 4 year follow-up. Increasing the interval to one year between the two doses of Gardasil® in 11–13 year-old girls has demonstrated similar levels of HPV-16 and HPV-18 antibodies to those observed in a three-dose Gardasil® regimen, one year after vaccination [58], [59].

A head-to-head trial of two doses of Cervarix® or Gardasil® six months apart in 9–14 year-old girls demonstrated higher antibody levels for Cervarix® than Gardasil® at one year following initial vaccination [60]. Two doses of Cervarix® also induced higher antibody levels than three doses of Gardasil®. These studies have demonstrated that dosing interval is an important determinant of immunogenicity.

Immunogenicity data for a single vaccine dose are limited and to date has been examined in clinical trial participants who did not complete protocol or in trials that ended prematurely. Although lower than the levels induced by two- and three-dose schedules, a single dose of Cervarix®, in 18–25 year-old women from the NCI-sponsored Costa Rican Vaccine Trial, induced detectable HPV-16 and HPV-18 ELISA antibody levels in all participants. These remained approximately 9-fold and 5-fold higher than natural infection levels, respectively, 4 years after initial vaccination [56]. In a Gardasil® trial in adolescents in India that was ended prematurely, only a fraction of recipients had HPV-16 and HPV-18 seropositive responses following one-dose vaccination as assessed with multiplex bead assays (49% and 58%, respectively) twelve months post-vaccination, with levels around the assay seropositivity cut-off, but these were reported as stable over a 36 month follow-up [57]. These preliminary data suggest that one dose of HPV vaccine may be sufficient to confer protective immunity against HPV. The clinical trials being conducted in Costa Rica (ClinicalTrials.gov Identifier: NCT03180034) and Tanzania (ClinicalTrials.gov Identifier: NCT02834637) will provide opportunities for a comprehensive head-to-head comparison of immune responses induced by Cervarix® and Gardasil®9, in the context of one- and two-dose regimens in young adolescents.

With the increasing number of trials relying on immunogenicity data to support new vaccine recommendations, evaluation of one-dose trials, and approval of new follow-on vaccines, there is a need for the critical assessment of the sensitivity, specificity, reproducibility and comparability of HPV immunogenicity assays. These assays are briefly described in the next section along with the role for International Standards and steps being taken to address gaps in availability of these key reagents.

## 3 HPV vaccine immunogenicity assays and considerations for single dose studies

Measures of the type-specific immune response to HPV after vaccination include both cellular as well as humoral markers. The HPV vaccines induce potent T and B cell responses both in animal models and in humans [61]. However, most attention has focused on the humoral responses given the proposed role that neutralizing antibodies have in protection against infection and subsequent disease [62]. Serology assays include those detecting antibodies (neutralizing assays and binding assays), as well as those measuring antibody binding strength (avidity). The cellular assays include assays for evaluation of T cell (CD4 and CD8 T cell responses) and of memory B cell responses. The remainder of this review will focus on the most commonly used serology assays in vaccine trials.

**Fig. 1:**
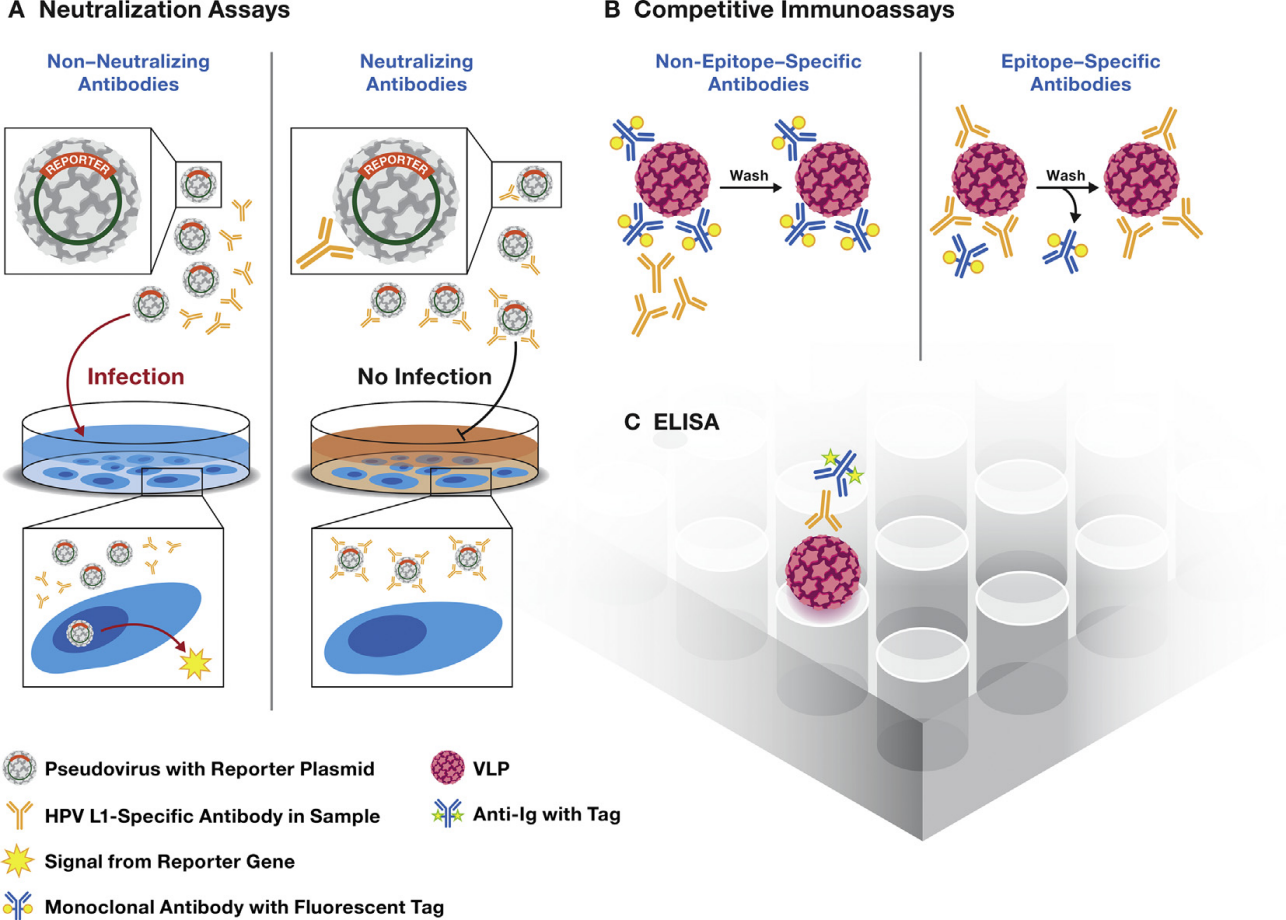
Three main types of assays have been used in the evaluation of antibody responses to HPV vaccines. (A) Neutralization assays, (B) Competitive immunoassays and (C) ELISA.

Three main serological assays have been used to evaluate HPV L1 VLP antibody responses to HPV L1 VLP vaccines in clinical trials (Fig. 1): (1) pseudovirus-neutralization assays [63], (2) competitive (epitope-specific) immunoassays [64], [65], [66], and (3) VLP-IgG binding assays [67]. Neutralization assays are considered the most relevant for measuring the biological activity of the antibodies and the WHO has suggested that such assays should be considered the reference standard for assessing protective antibodies induced by the vaccines [68]. Competitive immunoassays, such as the cLIA, estimate neutralizing activity by measuring competition of test serum with neutralizing monoclonal antibodies. In contrast, ELISAs detect all antibodies, regardless of neutralization ability. Most L1 VLP ELISAs detect IgG, but other immunoglobulin classes (such as IgA) or IgG subclasses can be targeted if the specificity of the secondary antibody is changed. The glutathione S-transferase (GST) fusion L1 immunoassays are similar to L1 VLP ELISAs in that they detect all binding antibodies of particular immunoglobulin class that is determined by the secondary antibody. However, GST fusion assays rely on an L1 protein target that is synthesized in bacteria and is not assembled into conformational VLPs [69]. The results of these assays generally correlate well, particularly in specimens with high antibody levels, suggesting that the dominant immune response to HPV vaccination is IgG against neutralizing epitopes [42], [67], [70], [71], [72], [73], [74].

The neutralization assays measure total immunoglobulins (IgM, IgA, and IgG) that exhibit *in vitro* neutralizing function (Fig. 1A). This assay, originally developed by Dr. Schiller’s laboratory at the National Institutes of Health [63], has been widely adopted. The assay makes use of pseudovirions that incorporate the minor (L2) capsid protein, as well as the L1 protein and an encapsidated reporter gene, such as secreted alkaline phosphatase (SEAP) or luciferase [63], [75], [76], [77], [78]. Following pseudovirion binding to and entry into target cells, the reporter plasmid is translocated to the nucleus and expressed. When neutralizing antibodies are present, pseudovirus infection is blocked with a concomitant reduction in reporter signal. Antibody levels are generally expressed as the reciprocal of the sample dilution resulting in an *a priori* level of inhibition (usually 50%). Since they are cell-based, neutralization assays tend to have a higher coefficient of variation than binding assays, are labor intensive, and costly. Generally, these have been difficult to adapt to a high-throughput format, although a 384-well format has been described [78]. Most formats of the assay are not amenable to multiplexing, but a recent report achieved a three-plex assay using three different reporter genes expressing different fluorescent proteins [77].

The cLIA assay requires labeled type-specific monoclonal antibodies and conformationally intact VLPs (Fig. 1B). Antibodies in the serum sample bind to VLPs and prevent binding of the labeled monoclonal antibody, thereby reducing signal. Concentrations are generally based on a standard curve. The assay measures antibodies of all immunoglobulin classes but, in principle, only to one epitope. Thus, it has high specificity, but results may underestimate the total functional antibody levels. The assay is epitope specific, rapid, readily multiplexed and amenable to high-throughput platforms. Vaccine trials sponsored by Merck used a competitive multiplex bead array assay [65], [66].

ELISAs also require conformationally intact VLPs and they measure both neutralizing and non-neutralizing antibodies, recognizing both conformational and non-conformational epitopes (Fig. 1C). VLPs are bound to a solid surface (beads or wells) and antibodies in the sample bind directly. Bound antibody is detected with labeled secondary antibodies directed against a specific Ig class, usually anti-IgG. The label can be an enzyme, an affinity label or a fluorescent molecule. L1 VLP ELISAs for type-specific IgGs have been used by GlaxoSmithKline and several research labs as the main assay in a number of immunogenicity trials [19], [20], [21], [56], [67], [79]. ELISAs for many other targets have been widely used, so the format is familiar to laboratories. The format is adaptable to multiplexing with bead arrays [70], [72], [80] or multi-spot wells [73], fast and amenable to high-throughput testing. However, only one immunoglobulin class is detected at a time and both neutralizing and non-neutralizing antibodies will generate a signal.

The overall advantages and disadvantages of the three broad classes of serology assays are summarized in Table 2. Both qualitative (positive/negative) and quantitative results (magnitude of antibody levels) are important. The quality of all these assays is dependent on the quality of the VLPs, pseudovirions and QC procedures [68]. Validated critical reagents and assay standards are not available commercially and therefore must be prepared and validated by the laboratory. Strict adherence to standard operating procedures for production and quality control of these reagents is required, monitoring purity, concentration and type-specific conformation for each lot. When the immunogenicity of two different vaccine formulations is being compared, the use of VLP antigens produced in an expression system that is different from that used to produce the vaccines would be preferable to avoid potentially favoring one formulation over another [18], [21]. Assay reproducibility should be monitored with the use of high- and low-antibody titer control sera in every run and trends monitored. Cut-off values can be challenging to determine, and although most laboratories base the value using a panel of virginal sera, such as children’s sera, there are critical differences between cut-off determinations used in different laboratories and assays. Currently there are no guidelines on methods to establish serostatus cut-offs which has a major influence on seropositivity rates. Therefore, establishing uniform criteria for cut-off determinations of seropositivity will be particularly critical for one-dose trials where lower levels of antibodies are anticipated.

**Table 2:** Three main assay types used in monitoring HPV vaccine immunogenicity and their advantages and disadvantages.

	Advantages	Disadvantages
Neutralization assay	• Measures function closest to presumed mechanism of protection • All immunoglobulin classes are detected	• Requires pseudovirions for each type • Requires cell culture and time for cells to grow • Time-consuming and labor intensive • Limited ability to multiplex • Higher coefficients of variation


Competitive immunoassay	• Detects neutralizing antibodies • Easily multiplexed with bead arrays (e.g. Luminex) • Rapid, high throughput • All immunoglobulin classes detected	• Only a subset of total neutralizing antibodies detected • Requires type-specific neutralizing monoclonal antibodies • Mulitplexing requires compromise between selecting dominant epitope and retaining type specificity


Enzyme linked immunosorbent assay	• Fast, high throughput • Familiar assay format • Amenable to multiplexing (bead arrays or multispot wells)	• Detects one immunoglobulin class (IgG or IgA), determined by secondary antibody • Non-neutralizing binding antibodies can be detected

Because different laboratories have used different assays for evaluation of immunogenicity to the HPV vaccines and key reagents are prepared differently in each laboratory, inter-laboratory assay standardization has been quite challenging. In 2006, the Bill and Melinda Gates Foundation funded a WHO Global HPV Laboratory Network whose main activities were to work out systems for international comparability of HPV ELISA assays for HPV-16 and HPV-18 [81], [82], [83]. A series of international collaborative studies proved the basic principles of comparability [82], [83], [84]. The serology assays in use were found to generate sufficiently similar results, such that a simple use of the same international reference standard in all laboratories gave highly comparable data [83]. Such reference sera are formally established as International Standards (IS) that define the International Unit (IU) of HPV antibodies and can be ordered from the National Institute of Biological Standards and Controls (NIBSC, Potters Bar, UK). A simple mathematical calculation called the parallel line method can be used to compare the crude results from a dilution series of the serum to be tested with the reference serum to find the antibody level [83]. Ideally, serology assay results should be reported in IU to facilitate direct comparison of results. The laboratory step-by-step protocol for HPV-16 and HPV-18 assays is published in the WHO HPV Laboratory Manual, which also details the protocols of two common serology methods found to be robust and transferable (the pseudovirion neutralization assay and the VLP ELISA assay) [68].

In addition to concentrations, antibody quality can be evaluated in terms of binding strength of antibodies to VLPs, or avidity. Only a few studies have examined avidity in samples from HPV vaccine trials [18], [57], [85], [86]. Avidity assays use the ELISA format but add a dissociating step with chaotropic agents such as ammonium thiocyanate, sodium thiocyanate or guanidine hydrochloride. Generally, the ratio of antibodies detected after dissociation to those detected without dissociation is used to calculate an avidity index. Care must be taken when selecting chaotropic agents and concentrations to ensure that the dissociation step does not introduce significant conformational change to the VLPs. Several assay variations have been reported [87], [88], [89], [90] but no standard avidity assay or interlaboratory comparisons have been performed to date. Affinity maturation and increases in avidity have been shown with increasing doses and time following vaccination [18], [57], [89], [90]. Although lower than that observed in three-dose recipients, high avidity levels indicative of antibody affinity maturation have been observed in one-dose recipients of Cervarix® and Gardasil® [57], [85]. The importance of antibody avidity in antiviral protection has been shown experimentally for some viruses, however, the significance of avidity in protection against HPV infection is not yet known [91]. In low antibody level situations, such as may be encountered with one-dose schedules, this could be an important parameter to monitor.

Robust and standardized serological assays are critical for current and future HPV vaccine evaluation and may help identify immune correlate(s) of protection and provide early indications of potential efficacy of new vaccine products, such as biosimilars. For closely related vaccines, immunogenicity data may support regulatory approval, accelerating vaccine implementation.

Based on long-term efficacy data, HPV vaccines are expected to induce sufficiently high levels of immunity to protect against infection and subsequent disease. However, low levels of antibodies are expected to be induced by single dose regimens, in particularly for HPV-18 in Gardasil® and Gardasil®9 recipients as well as for HPV-45 in Gardasil®9 recipients, based on long-term antibody persistence data and the data published in reduced dose schedules [19], [49], [92], [93]. Thus, efforts to increase serology assay sensitivity at the lower end may be required to support immunobridging trials for single dose vaccines. Results from mouse intravaginal challenge systems suggest that the levels required for *in vivo* protection are estimated to be orders of magnitude lower than levels detected *in vitro* by ELISA or neutralization assays [75], [94], suggesting that strategies for improvement in detectability of HPV antibody responses *in vitro* to match *in vivo* protection are warranted.

It is clear that immunogenicity assessment in one-dose vaccine studies will be challenging. The importance of the immunologic data from these trials requires that the data from these assays be reliable and interpretable. This means that testing must be done with methods that allow reporting results in terms of directly comparable International Units. International Standards are still required for the additional 5 oncogenic types included in Gardasil®9. A newly launched HPV Serology Standardization project, co-funded by the Bill and Melinda Gates foundation and the US National Cancer Institute, is addressing critical needs for global harmonization of HPV serology testing in clinical trials of HPV prophylactic vaccines. The project plans to identify additional secondary standards for the types included in current vaccines, provide critical reference reagents (VLPs), and develop testing guidelines, all of which will be made available to the HPV serology community. This builds on previous efforts of the WHO HPV LabNet and will be done in parallel with ongoing WHO and NIBSC efforts for production of International Standards for the additional HPV types included in Gardasil®9.
